# The Role of Satellites in the Evolution of Begomoviruses

**DOI:** 10.3390/v16060970

**Published:** 2024-06-17

**Authors:** Anupam Varma, Manoj Kumar Singh

**Affiliations:** Advanced Centre for Plant Virology, Division of Plant Pathology, Indian Agricultural Research Institute, New Delhi 110012, India; mksingh.acpv@rediffmail.com

**Keywords:** alphasatellites, betasatellites, deltasatellites, economic losses, whitefly-transmitted diseases, global warming

## Abstract

Begomoviruses have emerged as destructive pathogens of crops, particularly in the tropics and subtropics, causing enormous economic losses and threatening food security. Epidemics caused by begomoviruses have even spread in regions and crops that were previously free from these viruses. The most seriously affected crops include cassava; cotton; grain legumes; and cucurbitaceous, malvaceous, and solanaceous vegetables. Alphasatellites, betasatellites, and deltasatellites are associated with the diseases caused by begomoviruses, but begomovirus–betasatellite complexes have played significant roles in the evolution of begomoviruses, causing widespread epidemics in many economically important crops throughout the world. This article provides an overview of the evolution, distribution, and approaches used by betasatellites in the suppression of host plant defense responses and increasing disease severity.

## 1. Introduction

Begomoviruses have emerged prominently during the last four decades as the most successful group of devastating plant viruses, affecting a wide range of dicotyledonous crops worldwide, particularly in the tropics and subtropics, causing enormous economic losses and threatening food security. The estimated economic loss due to begomoviruses in the 1990s was USD 1.3–2.3 billion for cassava in Africa, USD 5 billion for cotton in Pakistan, USD 300 million for grain legumes in India, and USD 140 million in Florida, USA, for tomato alone [1]. Despite efforts to contain begomoviruses, they continue to emerge and re-emerge in diverse crops and regions [2,3,4,5,6,7].

In the early 1960s, about 27 whitefly-transmitted viruses (WTVs) were known to infect crop plants and weeds [8]. Cassava mosaic, cotton leaf curl, bean golden mosaic, cowpea golden mosaic, and yellow mosaic of legumes were the most serious whitefly-transmitted diseases (WTDs). Cassava mosaic and cotton leaf curl diseases are classical examples of the emergence of diseases by the introduction of new crops or genotypes susceptible to indigenous viruses occurring in new regions [2,9]. Cassava was introduced to Africa from South America in 1558, and the mosaic disease of cassava (CMD) was first recorded in East Africa in 1894; since then, CMD has spread to all cassava-growing areas in Africa [10]. Cassava was also introduced to India, where CMD became a major threat to the cultivation of cassava in the 1940s [1]. Cotton leaf curl disease (CLCuD), perhaps the most destructive WTD as almost no yield is obtained from the affected plants, was first observed in Nigeria in 1912 in cotton varieties introduced from America to improve cotton production in Africa [9]. However, CLCuD remained confined to Nigeria and Sudan [8]. In the 1960s, CLCuD also emerged in a small pocket in Pakistan and spread fast due to the introduction of the susceptible variety S12 in the late 1980s [11]. Around the same time, CLCuD appeared in India in some susceptible accessions of *Gossypium barbadense*, and within a few years, the disease appeared in alarming proportions in commercial cotton fields growing *G. hirsutum* in the northwest region of India [1]. CLCuD is shown to be caused by monopartite begomoviruses that require betasatellites for symptom expression [12,13].

Begomoviruses and their satellites are efficiently transmitted by polyphagous whitefly (*Bemisia tabaci*). In the 1980s, the appearance of a B-biotype whitefly capable of feeding on a wider range of hosts in the Americas resulted in the emergence of the previously unreported tomato mottle virus (ToMoV) in Florida and the re-emergence of tomato golden mosaic virus (TGMV) in Brazil [1], whereas *B. tabaci*, prevalent in the northwest of the Indian sub-continent, are known to be polyphagous with a wide host range; this may explain the high incidence of the diseases caused by begomoviruses in the region. Up to 1970, the population of whitefly was active during the wet season from mid-June to early October, and the crops grown before or after the wet season remained free from begomovirus infections. However, with the increase in irrigation and a gradual rise in global temperatures, the population dynamics of *B. tabaci* gradually changed, and they remained active throughout the year, resulting in the emergence of diseases caused by begomoviruses in crops that were previously free from such infections [1]. The global-warming-led increase in the *B. tabaci* population and its spread have also resulted in the emergence of begomovirus-induced diseases in parts of Europe that were not affected by such diseases earlier [14].

Begomoviruses are transmitted by whitefly in a semi-persistent circulative manner without replication in the vector [15]. However, the recent demonstration of replication of tomato yellow leaf curl China virus (TYLCCNV) in the salivary glands of whitefly using the vector DNA synthesis machinery [16] has introduced the possibility that some other begomoviruses may also be replicating in the vector whitefly. It would be interesting to know whether TYLCCNV-associated betasatellites also replicate in the vector whiteflies, which would add another dimension to the epidemiology of begomoviruses.

The genome of begomoviruses is either monopartite or bipartite and consists of circular ssDNA molecules encapsidated in twinned (geminate) particles. The begomoviruses spreading in the New World are mostly bipartite, whereas those spreading in the Old World have both bipartite and monopartite genomes. Tomato leaf curl virus (ToLCV), originating from Australia, was the first begomovirus shown to have a monopartite genome. ToLCV was also found to be associated with an ssDNA satellite molecule, which had no apparent effect on either viral replication or on symptom development [17]. Soon, satellite ssDNA molecules, about half the size of helper virus genomes, were found to be associated with other monopartite begomoviruses, including Ageratum yellow vein virus (AYVV) and cotton leaf curl begomoviruses causing CLCuD, which require betasatellites for symptom expression, and were shown to affect the replication of their helper begomoviruses and symptom development in some host plants [16,17]; these satellite molecules were previously designated as DNA β, but now, they are classified as betasatellites.

The betasatellites associated with CLCuD are not specific; for example, the association of the cotton leaf curl Multan betasatellite has been detected in association with eight different CLCuD-causing begomoviruses [9], and eight distinct betasatellites have been detected to be associated with tomato leaf curl disease [18], indicating that CLCuD complexes are still evolving. Both AYVV and cotton leaf curl Multan virus (CLCuMV) are also associated with other circular ssDNA molecules, now referred to as alphasatellites, which are about half the size of begomovirus genomes and, unlike betasatellites, are capable of self-replication in host plants but require a helper begomovirus for movement in plants as well as insect transmission [12,19]. Monopartite begomoviruses and the associated satellite molecules are mainly distributed in the Old World; some sporadic reports of their occurrence in the New World could be due to the inadvertent introduction of infected plants in the region [20]. In the Indian subcontinent, betasatellites are also associated with some bipartite begomoviruses [18,21,22]. Recently, satellite-like molecules have also been detected in association with bipartite begomoviruses causing CMD in Africa [23], indicating that the association of satellites with bipartite begomoviruses may also be common.

The genome of bipartite begomoviruses is designated as DNA A and DNA B; DNA A of both bipartite and monopartite begomoviruses contains one or two, virion-sense and four complementary-sense open reading frames (ORFs), whereas DNA B has one virion-sense and one complementary-sense ORF. They share a common region containing the origin of replication and the regulatory regions for bidirectional transcription. DNA A encodes proteins for replication, control of gene expression, and coat proteins. The DNA B component encodes two proteins involved in the intercellular movement of the virus in host plants and symptom development [1,6]. It is interesting that the ORF AV2 of DNA A is found only in begomoviruses occurring in the Old World [24]; it may have made DNA B redundant, resulting in the evolution of monopartite begomoviruses and its role taken over by the satellites associated with begomoviruses in the Old World.

Begomoviruses are known to cause serious diseases like leaf curl in cotton, okra, tobacco, and tomato and yellow mosaic diseases in grain legumes (such as cowpea, French bean, mung bean, and others) in the tropics for a long time, but since the Green Revolution (GR) period, the number of begomoviruses and their host range has increased enormously. From <30 WTVs causing diseases in crop plants and weeds [8] in the pre-GR period, the total number of characterized begomoviruses increased to about 100 species at the turn of the century [25], and to 445 species in 2023, as identified by the International Committee on Taxonomy of Viruses (ICTV); begomoviruses represent the largest number in the entire virosphere.

## 2. Satellites Associated with Begomoviruses

Satellite viruses and satellite RNAs that depend on helper viruses for replication, encapsidation, and transmission are unique to plant viruses. The term satellite virus was first used by Basil Kassanis in 1962 for the 17 nm diameter particles associated with tobacco necrosis virus. Since then, a large number of other satellite viruses and satellite nucleic acids associated with different groups of viruses, including begomoviruses, have been described [26].

Three types of circular ssDNA satellite molecules, designated as alphasatellites, betasatellites, and deltasatellites, are associated with diseases caused by begomoviruses. Of the three groups of satellites, betasatellites play a significant role in increasing the virulence of begomoviruses, whereas alphasatellites and deltasatellites do not seem to play any noticeable role in viral pathogenesis. Betasatellites are also shown to impact crop productivity; in cotton, the seed yield losses caused by CLCuD are determined by the level of betasatellite DNA rather than the viral DNA level [27]. The associated satellites replicate via a rolling-circle replication mechanism. Apart from alphasatellites, betasatellites and deltasatellites, a new group of non-coding small (<1 kb) DNA molecules, named gammasatellites, have also been reported from Guatemala and Puerto Rico [28], showing that begomovirus complexes are continuing to evolve.

### 2.1. Alphasatellites

More than 40 species of alphasatellites, the Rep-encoding satellite molecules, are reported to be associated with begomoviruses [29]. Alphasatellites have the ability of autonomous replication, but they depend on the helper virus for cellular movement, encapsidation, and transmission by the vector whitefly. Alphasatellites were first identified in association with monopartite begomovirus infections in the Old World that are known to harbor betasatellites, and more recently in plants infected with bipartite begomoviruses [30]. Biological functions of alphasatellites await elucidation. Generally, alphasatellites are shown to attenuate disease symptoms and reduce virus accumulation [5,31], but the alphasatellites associated with Euphorbia yellow mosaic virus (EuYMV) were shown to increase disease severity and virus accumulation in plants co-inoculated with the helper begomovirus, and they reduced the ability of transmission by the vector [32]. In a recent study, betasatellites were shown to impair the ability of ToLCNDV to maintain cotton leaf curl Multan alphasatellites in mixed infection, suggesting their incompatibility [33].

### 2.2. Betasatellites

Betasatellites are mostly associated with monopartite begomoviruses and with some bipartite begomoviruses spreading in the Old World. Betasatellites need cognate helper viruses for replication, encapsidation, cellular movement, and transmission by the vector whitefly. However, some betasatellites have acquired the ability to trans-replicate with non-cognate begomoviruses, but replication is supported by their cognate helper viruses better than non-cognate helper viruses [34]. Pepper plants (*Capsicum annuum*) showing severe leaf curl symptoms were found naturally co-infected with tomato leaf curl Joydebpur virus (ToLCJoV) and tomato leaf curl Bangladesh betasatellite (ToLCBDB). Even in agroinoculation, the natural non-cognate interaction between ToLCJoV and ToLCBDB developed severe symptoms compared to the mild symptoms developed by cognate combination (ToLCJoV × ToLCJoB) in infected plants [35].

Analysis of the sequences shows that many betasatellites contain iteron-like elements homologous to those of their respective cognate helper begomoviruses, suggesting that betasatellites may have evolved by acquiring homologous iteron-like sequences for their efficient replication [34]. However, heterologous combinations can also increase the severity of symptoms, as observed in TYLCV overcoming the resistance of F1 hybrid tomatoes grown in commercial greenhouses in Israel in combination with cotton leaf curl Gezira betasatellite [36].

Analysis of the genomic sequences of nearly 120 identified species of betasatellites shows that their genomes have distinctive organization consisting of an adenine-rich region, a satellite conserved region, and a single ORF-encoding βC1 protein [5]. The βC1 protein plays an important role in symptom development and the suppression of innate host resistance; good progress has been made in elucidating the molecular mechanisms for overcoming the host resistance [5,27,28]. The most significant role of the βC1 protein is shown to interfere with the plant defense pathway, leading to the suppression of host plant defense responses. Multifaceted roles of the *βC1* gene have been very nicely illustrated by Gnanasekaran et al. [37]. Here, some selected examples are included to show the roles of betasatellites associated with monopartite and bipartite begomoviruses.

The *βC1* gene of cotton leaf curl betasatellite (CLCuB) associated with CLCuD encodes the pathogenicity protein responsible for symptom production through interaction of the G-box motif with host nuclear factors for efficient gene expression and replication of the satellite [38]. However, the CLCuB *βC1 gene*, in combination with bipartite tomato leaf curl New Delhi virus (ToLCNDV), replaces the movement function of viral DNA B by transporting the viral DNA A from the nucleus (the site of virus replication) to the plasmodesmata to facilitate intra- and intercellular movement of the viral DNA A [39]. In another betasatellite (tomato leaf curl Patna betasatellite) and non-cognate monopartite begomovirus (tomato leaf curl Gujarat virus) interaction, the βC1 protein influences viral pathogenesis by substantially reducing the helper virus accumulation by interfering with the ATP hydrolysis activity of the helper virus Rep protein, as the C-terminal region of βC1 protein interacts with the C-terminus of the Rep (RepC) protein coded by the virus [40]. Variations in begomovirus–betasatellite interactions have also been shown for cassava-infecting begomoviruses [41]. The ability of betasatellites to alter host resistance is the most important defense enabling the begomoviruses to evolve to become more infectious with improved transmission and imparting the ability to jump hosts.

In *Nicotiana benthamiana*, the interaction of the radish leaf curl betasatellite (RaLCB)-encoded βC1 protein with the oxygen-evolving enhancer protein 2 (PsbP) reduces PsbP-induced host resistance [42]. Recently, N-terminal acetylation of TYLCCNB-βC1 was shown to be critical for viral pathogenesis due to antagonistic crosstalk between *N*-acetylation and ubiquitination; in the absence of *N*-acetylation, the viral symptoms are attenuated [43]. Near-infrared (NIR) light confers antiviral immunity in plants by positively regulating phytochrome-interacting factor 4 (PIF4)-activated RNAi, but in plants co-infected with begomovirus and betasatellite, βC1 protein inhibits PIF4 transcriptional activity by disrupting PIF4 dimerization, ultimately facilitating virus infection [44].

Recently, a novel protein βV1 was shown to be encoded by several betasatellites [45]. βV1 protein helps in the rapid replication of viral genomes and contribute to symptom development, but the molecular basis of βV1-mediated regulation is not known [5]. The βV1 ORF is present in about 40% of the reported betasatellite sequences. Plants co-infected with TYLCCNV and TYLCCNB became incapable of expressing *βV1* gene by mutation, developing reduced virulence and viral titers. The βV1 protein could also induce a hypersensitive response in *N. benthamiana* leaves [46]. These findings add to the complexity of plant–begomovirus–betasatellite interactions.

### 2.3. Deltasatellites

Deltasatellites are small, non-coding satellite ssDNA molecules of about 0.7 kb that depend, like betasatellites, on the associated begomoviruses for replication, cellular movement, encapsidation, and transmission by the vector whitefly. Currently, 11 species of deltasatellites have been recognized by the ICTV. The first satellite found associated with ToLCV in Australia was a deltasatellite; initially, it was named ToLCV-Sat, and now it is known as tomato leaf curl deltasatellite (ToLCD). Five species of deltasatellites are associated with bipartite begomoviruses of the New World, and six with the Old Word begomoviruses [47]. Deltasatellites are known to reduce the replication of helper virus DNA and the development of symptoms induced by the helper begomovirus.

## 3. Role of Betasatellites in Transmission of Begomoviruses by *Bemisia tabaci*

Betasatellites are also shown to manipulate the interactions between vector whitefly, *B. tabaci,* and plants. Plants infected with TYLCCNV and its cognate betasatellite attract more vector whiteflies. It was shown that serine-33, a conserved phosphorylation site in betasatellite-encoded βC1 protein, suppresses the plant terpenoid-based defense, which promotes whitefly performance [48]. Extensive studies have demonstrated the role of jasmonic acid (JA) in begomovirus/betasatellite–plant–whitefly tripartite interactions in increasing the spread and severity of begomovirus infections in plants. TYLCCNV is shown to inhibit the JA-biosynthesis genes *FAD3* and *FAD7*, as well as the JA-regulated gene *PDF1.2* [4]. The TYLCCNB βC1 protein suppresses the synthesis of terpenoids, which improves the performance of the vector whiteflies [49,50]. Recently, βC1-NtSKP1 interaction was shown to interfere with the JA signaling pathway and attenuation of the plant JA defense responses [51]. These findings amply demonstrate that betasatellites interfere with plant defense responses to achieve successful infections by their associated begomoviruses.

In plants infected with tobacco curly shoot virus (TbCSV) and its betasatellite (TbCSB), the amount of TbCSB is higher at the initial stages of infection, and becomes constant at a later stage of infection. It was shown that a higher TbCSB/TbCSV ratio promotes transmission of the virus by the vector whitefly [52]. The efficiency of transmission of five different begomoviruses by the vector whitely was found to vary from virus to virus; similarly, in the vector transmission of beta- and alphasatellites, transmission efficiency by four different cryptic species of whiteflies have also been shown to vary [35].

## 4. Origin and Evolution of Satellites Associated with Begomoviruses

**Figure 1:**
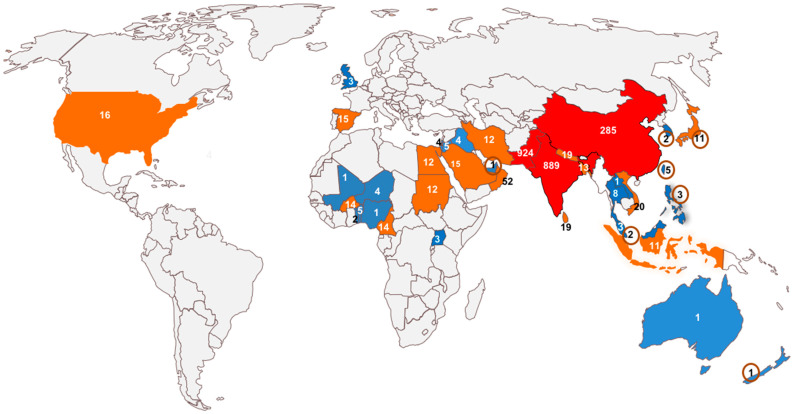
Global spread of betasatellites based on >2400 sequences submitted to date from different countries (indicated by numbers); the countries that have submitted <10 sequences are shaded blue, those that have submitted <100 sequences are shaded brown, and those that have submitted >100 sequences are shaded red; the countries shaded red are hotspots for betasatellites, representing 87% of the total sequences submitted so far; 11% of the sequences have been submitted by the countries shaded brown; and the remaining 2% of sequences have been submitted by countries shaded blue. The numbers in circles indicate the sequences submitted by the connected countries.

Begomoviruses seem to have acquired DNA satellites to gain an evolutionary advantage, as most betasatellites enhance the accumulation of viral DNA. The satellites, in general, may have originated from genetic elements that were once part of the viral genome but eventually lost their ability to replicate independently. Over time, these genetic elements may have become dependent on helper viruses to provide the necessary machinery for their replication and transmission. Alphasatellites are also known to be associated with some nanoviruses [53,54]. It is possible that some nanoviruses or their alphasatellites may have adapted to become encapsidated in the begomovirus coat protein for transmission by whitefly [55]. The evolutionary origin of betasatellites is not clearly understood, although it was suggested that these may be genetic remnants of some unidentified or extinct viruses [56]. However, the greater molecular diversity of betasatellites occurring in the Indian subcontinent and China (Figure 1) indicates that this region may be the center of origin of the betasatellites of begomoviruses.

Other factors, like genetic mutations, recombination events, and selection pressures, may also have driven the evolution of viral satellites by enhancing their ability to interact with the helper viruses and evade host defenses. Recombination events between different satellite and helper virus genomes can also contribute to the generation of new satellite variants. Selection pressures also plays a crucial role in the evolution of viral satellites. Begomovirus satellites seem to have efficiently exploited the resources of helper viruses to persist, spread, and disarm the host resistance by evading detection and/or manipulation of the host immune system. Mixed infections by monopartite and bipartite begomoviruses in the Indian subcontinent may have helped the development of the genetic diversity of begomoviruses associated with chili leaf curl disease (ChiLCD) and rapid adaptation of the viruses and their satellites to new environments, expanding the virus–host range [57,58]. The novel DNA satellites associated with bipartite begomoviruses in the New World were found to share some genetic features with betasatellites and also contain nucleotide stretches of begomoviral origin, presumably the remains of recombination events involved in their origin [59].

Field studies on begomoviruses are largely restricted to cropping systems, and limited attention has been paid to infections in non-cultivated plant species, although it is well known that virus infections in weeds are important for the viral evolution, spread, emergence, and re-emergence of new viral diseases. Weeds may also play an important role in the origin and evolution of satellite molecules. Recent studies have shown the association of satellite molecules with begomoviruses infecting weeds in diverse regions. Weed *Sinapis arvensis* is a reservoir of begomoviruses and associated betasatellites in Jordan [60]; *Solanum nigrum* is a host of tomato leaf curl Palampur virus and CLCuMB in Pakistan [61]; *Ageratum conyzoides* and *Amaranthus spinosus* are hosts of Ageratum yellow vein China virus and tomato leaf curl Java beta satellite in Indonesia [62]; and *A. conyzoides* is a host of Alternanthera yellow vein virus and tomato leaf curl betasatellite in Oman [63]. Earlier studies have shown the presence of multiple and recombinant betasatellites in weeds *Digera arvensis* and *Sonchus arvensis* [64,65]. These are just a few examples demonstrating that that coinfection of begomoviruses and their associated betasatellites may play important roles in the evolution of diverse begomovirus–betasatellite combinations.

## 5. Distribution of Betasatellites

The geographical distribution of betasatellites shows their occurrence mainly in Africa and Asia (Figure 1), and their maximum diversity seems to occur in the Indian subcontinent [37]. China, India, and Pakistan are home to over 87% of the betasatellites sequenced so far. The intensive continuous cropping system in these countries provides ideal conditions for the perpetuation of begomoviruses, their satellites, and the vector whiteflies. The association of betasatellites with diverse begomoviruses has also been reported from Africa, Europe, Middle East, and South East Asia, and to a lesser extent, from Australia and New Zealand.

In the New World, the WTDs are caused by bipartite begomoviruses, which have not been found to be associated with betasatellites. However, the introduction of monopartite begomoviruses, like TYLCV in Florida, USA, in the late 1990s through infected transplants [66], may have also introduced some TYLCV-associated betasatellites, but no betasatellites were detected in the region until 2018, when an unexpected outbreak of WTDs in okra occurred in Texas, USA [20]. The disease in okra was caused by a complex consisting of the monopartite cotton leaf curl Gezira virus (CLCuGV), cotton leaf curl Gezira alphasatellite (CLCuGA), cotton leaf curl Gezira betasatellite (CLCuGB), and the bipartite okra yellow mosaic Mexico virus (OkYMMV). These begomoviruses and their satellites may have been introduced in Texas through infected plants. The spread of these begomoviral complexes may be a threat to vegetable cultivation in the area [20]. However, there is no further information about their spread in Texas or other areas.

The WTDs affect a variety of crops in Brazil, as it provides ideal ecological conditions for the perpetuation of begomoviruses and their vector *B. tabaci*, but despite concerted efforts, betasatellites remain undetected in Brazil (Murilo Zerbini, personal communication). The genome of a novel monopartite begomovirus that infects non-cultivated *Galium mexicanum*, in Mexico, was found to have DNA-A similar to the New World bipartite begomoviruses, but contained iteron sequences found in monopartite begomoviruses from the Old World, highlighting the need for greater understanding of global virome ecology and evolution [67]. The introduction of monopartite begomoviruses, like TYLCV and CLCuGV and their associated betasatellites, in the region increases the chances of the acquisition of betasatellites by indigenous begomoviruses to enhance their virulence [68].

## 6. Discussion

Begomoviruses have emerged as serious plant pathogens due to their innate ability to counter plant defense and cause severe diseases. Their associated satellites, particularly betasatellites, have played a significant role in countering plant defense. The multifunctional βC1 protein encoded by betasatellites interacts with cellular proteins and modulates several cellular processes, especially targeting the innate immune system of plants. Useful information on the functional mechanisms of the βC1 protein and its interactions with helper viruses and host proteins has been generated [69]. The βC1 protein was shown to suppress plant defense mechanisms, such as post-transcriptional gene silencing, hormone-based defense systems, ubiquitine proteome degradation system, and autophagy [5,43]. Betasatellites associated with begomoviruses have obviously played a significant role in the evolution of begomoviruses by their ability to increase disease severity by disarming host resistance and improving transmissibility of the viruses by the vector whitefly. Understanding of the processes by which begomoviruses and their satellites disarm the host defense will be useful in developing strategies for the management of diseases caused by begomoviruses. For example, in transgenic *N. benthamiana* plants containing an RNAi construct, targeting the *βC1* gene of the betasatellites of cotton leaf curl Khokran virus and cotton leaf curl Multan virus considerably reduced the virus titer [70].

**Figure 2:**
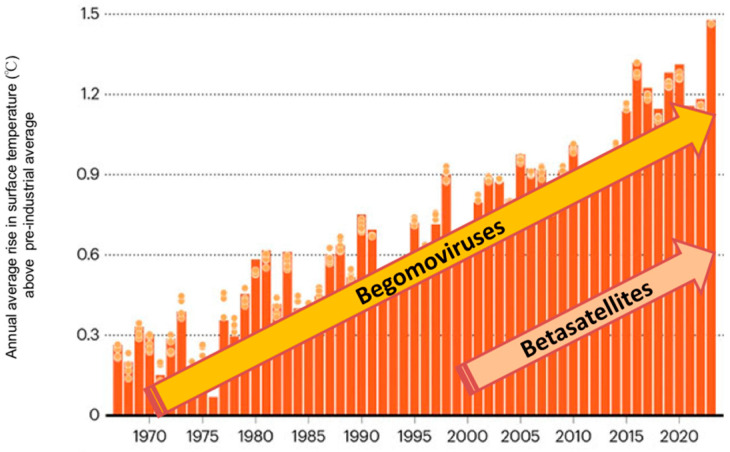
The average annual rise in surface temperatures above the pre-industrial average (1850–1900) since mid-1960s, coincides with the increase in the number of begomovirus species from <30 in 1970 to 445 species in 2023, and the number of betasatellite species from 7 in 2000 to 120 species in 2023 (modified from Nature Briefing, 15 January 2024).

The emergence of begomovirus–betasatellite complexes during the current century seems to be supported by global warming (Figure 2). Analysis of the rise in the surface temperature since the pre-industrial period (1850–1900) shows a consistent rise in temperatures, with 2023 being the hottest year on record. Past experience has shown that the rising temperatures have influenced *B. tabaci* population dynamics in north-west India, where *B. tabaci* used to be active only during the monsoon period (June to October) in the late 1970s; by the late 1990s, *B. tabaci* were active throughout the year and their population also increased manifold. This also resulted in the spread of begomoviruses to winter crops like potato [1]. The trend continues, further supported by the suppression of plant defense by the βC1 protein. Greater efforts are required to check increases in the *B. tabaci* population and development of their new biotypes.

Cropping systems seem to directly influence the distribution and evolution of begomoviruses and their satellites. Before the emergence of monopartite begomoviruses at the end of the last century, only bipartite begomoviruses were endemic in various parts of the world, except Africa, where leaf curl diseases have affected cotton for a long time. It is expected that some endemic monopartite begomovirus may have moved to newly introduced varieties of cotton to promote the cotton industry in Africa [9]. Interestingly, CLCuD did not occur in India until 1989, when it was first observed in a few plants of an introduced susceptible genotype, and soon the disease became a menace [1]. CLCuD may have played an important role in the evolution of monopartite begomoviruses and their satellites in the Indian subcontinent.

The global spread of betasatellites (Figure 1) shows China, India, and Pakistan to be hotspots for betasatellites, representing 87% of the total sequences submitted so far, with limited occurrence of betasatellites in other parts of the Old World. The New World is nearly free from betasatellites, although the areas must have been exposed to monopartite begomoviruses and their associated satellites through tomato transplants regularly used for plantings. TYLCV emerged in Florida in serious proportions, affecting nearly 100% of plants [66]. Interestingly, neither the virus nor the associated satellites could be established in North America. A recent report [20] on the occurrence of okra-associated begomoviruses and betasatellites in North America also showed that they seem to have been introduced with the planting material. It is expected that these have also not been established in the North American cropping systems. The hotspots for betasatellites in Asia may have developed due to the intensive cropping systems and more favorable environmental conditions, ideal for the perpetuation of begomoviruses and their vector *B. tabaci*.

## 7. Concluding Remarks

During the last two decades, betasatellites have propelled the emergence of begomoviruses as a serious threat to the production of a variety of crops in various ecosystems across the world. The ability of betasatellites to trans-replicate by several different begomoviruses and evolve highly virulent disease complexes by overcoming host resistance and increasing the host range has turned begomoviruses into the most destructive plant pathogens. The challenge is to develop effective strategies to minimize the damage caused by begomovirus complexes in various cropping systems around the world.
